# Results of the Pavlik harness when treating Ortolani-positive hips: predictors of failure and arthrographic findings

**DOI:** 10.1007/s11832-015-0666-8

**Published:** 2015-07-07

**Authors:** Pablo Vadillo, Carlos Alberto Encinas-Ullan, Luis Moraleda, Javier Albiñana

**Affiliations:** Department of Orthopaedics Surgery, Hospital Universitario Infanta Elena, Madrid, Spain; Department of Orthopaedics Surgery, Hospital Universitario HM Sanchinarro, Madrid, Spain; Pediatric Orthopaedics Unit, Department of Orthopaedics Surgery, Hospital Universitario La Paz, Paseo de la Castellana 267, 28046 Madrid, Spain; Department of Orthopaedic Surgery, Hospital Universitario Quirón, Madrid, Spain

**Keywords:** Hip, Ortolani, Pavlik, Failure, Arthrogram

## Abstract

**Background:**

Predictors of failure of the Pavlik harness in reducing and stabilizing an Ortolani-positive hip remain ‘unclear’. The purpose of this study is to investigate the success of the Pavlik harness when treating Ortolani-positive hips, to look for predictors of failure of the harness and to analyze the arthrographic findings among these failures.

**Materials and methods:**

The medical records of 39 consecutive patients with an Ortolani-positive hip treated initially with a Pavlik harness were reviewed. Data regarding birth order, problems during pregnancy, presentation at birth, delivery, family history of DDH, gender, side involved, bilaterality, onset of treatment, problems related to use of the harness, and time until the harness reduced and stabilized the hip or was abandoned because of a failure were recorded. The presence of plagiocephaly, torticollis or foot deformity was also noted. We looked for predictors of failure among these aspects and report the arthrographic findings of the failures.

**Results:**

The mean age when the harness was started was 16.7 days. The mean time until success or failure of the harness in reducing and stabilize the hip was 18.5 days. There were 8 (20.5 %) failures. Multigravida (*p* = 0.026) and foot deformity (*p* = 0.023) were associated with failure of the harness. On the other hand, problems during pregnancy (*p* = 1), presentation at birth (*p* = 0.078), c-section (*p* = 0.394), family history of DDH (*p* = 1), gender (0.313), torticollis (*p* = 1), bilaterality (*p* = 1) and onset of treatment (*p* = 0.485) were not associated. Arthrographic abnormalities were found in all failures.

**Conclusion:**

The Pavlik harness failed to reduce and stabilize the hip in 20.5 % of the newborns with an Ortolani-positive hip. Multigravida and foot deformity were statistically associated with failure of the harness. An anatomical obstacle for reduction was found in all hips with a harness failure. A more teratological than mechanical hip dislocation could be the reason for failure of the Pavlik harness.

**Level of evidence:**

IV, Retrospective case series.

## Introduction

The Pavlik harness has been widely used for the treatment of developmental dysplasia of the hip (DDH) since Arnold Pavlik first used it in 1944 [1]. Pavlik [1] reported an 85 % success rate in the reduction of dislocated hips. Lerman reported a 93 % success in reduction in Barlow-positive hips, and an 85 % success in Ortolani-positive hips. Success rates for the management of congenital dislocation of the hip by other researchers using the harness only in Ortolani-positive hips have ranged from 43−93 % [2–6].

Despite the safety of the Pavlik harness when properly used, there may be some complications derived from its inappropriate use [7]. Avascular necrosis of the femoral head (AVN), the most serious complication, usually occurs secondary to forced abduction or persistent use of the harness despite a failure to reduce the hip [7–9]. When treating a patient in whom the Pavlik harness is not achieving reduction and stabilization of the hip, the treating surgeon has to decide when to move to a more aggressive modality of treatment (arthrography followed by closed or open reduction), which has also been related to AVN [10, 11]. Although most clinicians agree in discontinuing the harness when failure to stabilize the hip is observed after 3–4 weeks of treatment [2, 4, 12, 13], it is still unclear as to the best moment to abandon the Pavlik harness or even if another modality of treatment should be the first choice in some cases.

The purpose of the study is to investigate the success of the Pavlik harness when treating Ortolani-positive hips, to look for predictors of failure of the harness and to analyze the arthrographic findings among failures.

## Materials and methods

Patients were identified through a computerized search by the ICD-9 code 754.30 among patients admitted to our institution between January 2006 and October 2010, and by checking the pediatric orthopedic outpatient clinic medical reports during the same period of time. Ninety-three patients were identified, including patients who underwent an Ortolani-positive maneuver by a senior pediatric orthopedic surgeon and who were initially treated with a Pavlik harness. In clinical examination, a hip was classified as ‘Ortolani-positive’ if the femoral head resided outside the acetabulum at rest but could be reduced into the acetabulum using the Ortolani maneuver [4, 14]. We excluded from this study patients with dislocable hips (Barlow-positive hips), neuromuscular disease, arthrogryposis, teratologic dislocation or non-reducible dislocation. The final group consisted of 39 patients. Medical records were retrospectively reviewed.

Data regarding sex, side of pathology, bilateralism, presentation at birth, age at birth, number of pregnancy, problems during pregnancy, limitation of abduction, Galeazzi sign, torticollis, foot deformity, family history of DDH, age at the time of diagnosis, age at the beginning of treatment with the Pavlik harness, harness failure, time of harness discontinuity, open or closed reduction in case of failure of harness and the presence of any complication were noted.

Clinical assessment consisted of a weekly visit in which the harness position was evaluated and the stability of the hip was recorded. Whenever the hip became stable during the first 3 weeks, we performed an anteroposterior radiograph of the pelvis to confirm reduction. In this group of patients the harness was maintained 23 h a day until the acetabular index normalized. In those cases where the harness failed to reduce and/or stabilize the hip within the first 3 weeks, the harness was discontinued and an arthrogram was performed under conscious sedation. If a congruous reduction was possible by closed maneuvers and the hip remained stable without force abduction, a spica cast was applied. Adductor tenotomies were performed in case of excessive tension with hip abduction. If the arthrogram showed any obstacle for reduction, an open reduction was performed at 6 months of age followed by immobilization in a spica cast for 3 months. Failure was defined as the inability of the harness to achieve or maintain a concentric reduction. Ultrasonographic screening was introduced in our institution in 2000 but it was not routinely used until 2010.

All arthrograms were reviewed and described by two pediatric orthopedic surgeons. The presence of medial pooling of the contrast (>2 mm of dye between the femoral head and the acetabulum), an inverted or everted labrum, a rose thorn sign, an interposed transverse ligament, hypertrophy of the ligamentum teres, a psoas shadow or an hourglass sign were noted.

Quantitative variables were described using average, median and standard deviation. Correlations of quantitative variables were studied by the Mann–Whitney *U* test. Qualitative variables were described using absolute and relative frequencies expressed as a percentage, as well as box-plot graphs. The chi-squared test or the Fisher’s exact test was used to analyze frequencies between qualitative variables. Time to failure was estimated using the Kaplan–Meier method and the log-rank test. A significant difference was defined as *p* < 0.05.

## Results

Demographic data are shown in Table 1. Data related to pregnancy and delivery are shown in Table 2. There were two problems (5.2 %) during pregnancy—oligohidramnios and premature rupture of membranes. Findings during physical examination are reported in Table 3.

**Table 1:** Demographic data

Age at diagnosis	16.7 days
Side involved	
Right	7 (18 %)
Left	14 (36 %)
Bilateral	18 (46 %)
Sex	
Males	6 (15 %)
Females	33 (85 %)
Female: male Index	5.5
Family history of DDH	3 (8 %)

**Table 2:** Obstetric data

Number of pregnancy	
First pregnancy	29 (74 %)
Second pregnancy	7 (18 %)
Third pregnancy	3 (8 %)
Presentation	
Cephalic Breech	28 (72 %) 11 (28 %)
Delivery	
Normal	27 (69 %)
C-section	12 (31 %)
Problems during pregnancy	2 (5 %)

**Table 3:** Physical examination

Galeazzi sign	3 (8 %)
Hip abduction limited	8 (20 %)
Congenital torticollis	1 (3 %)
Plagiocephaly	1 (3 %)
Foot deformity	8 (20 %)
Calcaneovalgus foot	5 (13 %)
Metatarsus adductus	3 (8 %)

The harness was successful in reducing and stabilizing the hip in 31 patients (80 %). The average time for reduction and stabilization of the hip was 18.5 (4–34) days.

The harness failed in 8 patients (20 %). When looking for factors related to failure of the harness, only a multigravida pregnancy (*p* = 0.026) and the presence of foot deformity (*p* = 0.001) were associated. The harness failed in two of the three third-pregnancy cases and in three of the seven second-pregnancy cases; however, it only failed in three of the 29 (10 %) first-pregnancy cases. There was a harness failure in four of eight cases with foot deformity, whereas there were only four failures among the 31 cases (13 %) without foot deformity. Problems during pregnancy (*p* = 1), presentation at birth (*p* = 0.078), cesarean section delivery (*p* = 0.394), family history of DDH (*p* = 1), gender (0.313), torticollis (*p* = 1), bilateralism (*p* = 1), harness malposition (*p* = 1) and age at diagnosis (*p* = 0.485) were not associated with failure of the harness. Data regarding the 8 patients with a failure of the Pavlik harness are shown in Table 4.

**Table 4:** Patients with a failure of the Pavlik harness

	No. of pregnancy	Weight at birth (g)	Presence of foot defomity	Age at diagnosis (days)	Presentation at birth	Cesarean section	Family history of DDH
Patient 1	1	3,480	No	34	Cephalic	No	No
Patient 2	1	3,550	No	3	Cephalic	No	No
Patient 3	3	3,000	Yes	1	Cephalic	No	No
Patient 4	2	4,120	Yes	2	Cephalic	No	No
Patient 5	2	3,300	Yes	3	Cephalic	No	No
Patient 6	3	3,960	No	2	Cephalic	Yes	No
Patient 7	2	3,200	Yes	2	Cephalic	No	No
Patient 8	1	3,540	No	70	Cephalic	No	No

**Fig. 1:**
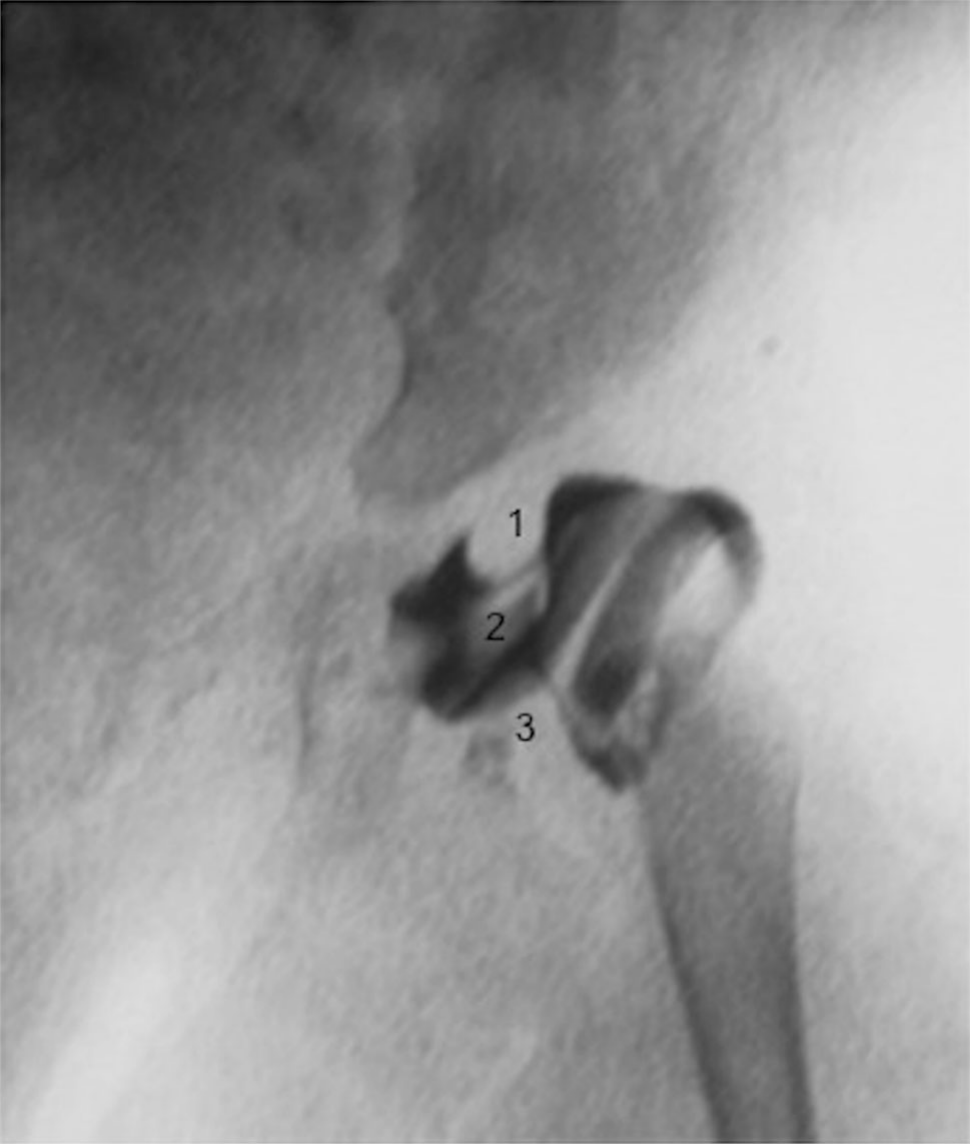
Arthrogram of a Pavlik harness failure that shows obstacles for reduction (*1* an inverted labrum, *2* ligamentum teres, *3* psoas tendon)

We performed an arthrogram in the 8 cases with a harness failure. Results are shown in Table 5. An incongruous acetabulum and/or unstable hip reduction were found arthrographically in all failures. We described a medial pooling of the contrast in all cases, an absent rose thorn sign in six cases, an hourglass sign in one case, a psoas shadow in four cases and an interposed transverse ligament in five cases (Fig. 1). The labrum was abnormal in seven cases, inverted in three cases and everted in four cases. The ligamentum teres was hypertrophied in four cases.

**Table 5:** Arthrographic results

	Patient 1	Patient 2	Patient 3	Patient 4	Patient 5	Patient 6	Patient 7	Patient 8
Medial pooling	Yes	Yes	Yes	Yes	Yes	Yes	Yes	Yes
Rose thorn sign	Absent	Absent	Absent	Absent	Present	Absent	Absent	Present
Inverted/everted labrum	Everted	Inverted	Everted	Inverted	Everted	Inverted	Inverted	Not able to define
Interposed transverse ligament	No	Yes	No	Yes	No	No	Not able to define	Yes
Hourglass sign	No	No	No	No	No	No	Yes	No
Psoas shadow	Yes	No	Yes	No	Yes	Yes	No	No
Hypertrophic ligamentum teres	Yes	No	Yes	No	Yes	Yes	No	No

We performed two closed reductions and six open reductions in the 8 patients with a Pavlik harness failure. We achieved a concentric and stable reduction in these 8 patients. Latest radiograph showed an acetabular index >30 degrees in two cases—36 and 38 degrees, respectively. There were two cases of avascular necrosis of the femoral head, both of them after an open reduction.

## Discussion

When managing a patient with a dislocated hip, the aim is to reduce and stabilize the hip by conservative means [4]. Pavlik defined the use of the harness as a functional treatment where active and spontaneous movements of the hip joint are allowed. Non-active forces besides gravitational forces are used for reduction and movement of the child and tend to reduce the hip providing a maximum opportunity to attain reduction with minimum risk of avascular necrosis. However, success rates of the harness for the management of Ortolani-positive hips range from 43−93 % [2–6]. Despite the safety of the Pavlik harness when properly used, there may be some complications derived from its inappropriate use [7]. Moreover, although the common approach is to perform a closed or open reduction and immobilization with a spica cast in those hips that remains unstable, some authors have proposed to change the Pavlik harness for a semi-rigid hip abduction brace [4].

In our series, the Pavlik harness succeeded in 80 % of the Ortolani-positive hips. These results are consistent with previous studies [4, 5] and better than other published series [2, 3, 6]. Swaroop et al. [4] described an 85 % success rate of the Pavlik harness in 52 Ortolani-positive hips (39 patients) when a protocol similar to ours was used. These authors described an increase in the success rate from 85 % to 93 % when patients with hips that remained unstable at 3 weeks of treatment in the Pavlik harness were transitioned to a semi-rigid hip abduction brace [4]. Although they do not report any cases of AVN of the femoral head, we are concerned about the possibility of developing AVN of the femoral head if we force abduction with a rigid device. Thus, we prefer to perform a hip arthrogram followed by closed or open reduction if the Pavlik harness fails to reduce and stabilize the hip. Arthrography is a proven method for identifying structures where it may be difficult to attain reduction and helps us to decide when to perform a closed or open reduction [15, 16]. Whether Swaroop’s protocol with a semi-rigid device or a closed/open reduction under general anesthesia leads to a higher incidence of AVN of the femoral head remains debatable.

In our series, we found that multigravida and the presence of foot deformity were statistically associated with failure of the harness. First pregnancy is a well-known risk factor for DDH probably because of the mechanical effect of the tight, non-stretched mother’s abdominal wall and uterus, which tends to compress the fetus much more than in later pregnancies [17]. Our higher rate of failure of the harness among non-first pregnancies could be explained by the fact that hip dislocation could be a consequence, in these cases, of a more severe anatomical abnormality. Associated foot deformities might be another manifestation of such factors, which would confirm our hypothesis. The idea of an anatomical explanation for the Pavlik harness failure in infants with a positive Ortolani examination is concordant with White et al. [6]. They found, using ultrasound, that an inverted labrum and a lateral and/or superior femoral head displacement were related with the failure of the Pavlik harness. However, they did not find first pregnancies or bilateralism to be statistically related with this prediction.

It still remains controversial as to whether age at the beginning of treatment is a risk factor for failure of the harness [2, 5, 12]. We did not find a statistical association probably because all patients were treated during the first months of life. Suzuki et al. [18] considered that the rate of successful reduction was more dependent on the severity of the dislocation rather on the age of the patients at the beginning of treatment. The authors considered that factors that prevent from reduction such as a tight ilio-psoas tendon, a capsular isthmus or intra-articular obstacles, might be present in the wide distance between the femoral head and the acetabulum, and that they could be responsible for the poor rate of success in severely dislocated hips [18].

At our institution we systematically perform a hip arthrogram when there is a failure of the harness. We observed arthographic obstacles for reduction in all failures, which were confirmed intraoperatively [19, 20]. In our opinion, these findings suggest that Ortolani-positive hips that fail to reduce and stabilize within a Pavlik harness present a more severe distortion of hip joint anatomy. We believe that a more ‘teratological’ than mechanical hip dislocation could be the reason for the failure of the Pavlik harness in these cases.

In conclusion, the Pavlik harness obtains a high rate of success when treating Ortolani-positive hips. We found multigravida and the presence of foot deformity to be statistically associated with failure of the harness and no other manifestations of mechanical etiology. Hip arthrograms showed obstacles preventing reduction in all failures.
